# Effects of HPV16E6 transfection on the biological behavior of Eca109 and Eca9706 cells

**DOI:** 10.3892/ol.2021.12552

**Published:** 2021-02-15

**Authors:** Xin-Dan Kang, Yong Zheng, Wei-Gang Chen, Min Cheng, Di Zhang

Oncol Lett 15: 1646-1654, 2018; DOI: 10.3892/ol.2017.7469

Subsequently to the publication of this paper, an interested reader drew to the authors’ attention that the ‘Negative control’ and ‘HPV16-E6’ data panels in Fig. 7 for the Eca9706 cell line appeared to contain strikingly similar data, such that they may have been derived from the same original source. The authors re-examined their original data, and were able to demonstrate to the Editorial Office that this figure had been assembled incorrectly.

The revised version of Fig. 7, featuring the correct data for the abovementioned ‘Negative control’ and ‘HPV16-E6’ data panels, is shown opposite. The authors regret the inadvertent errors that were made during the compilation of Fig. 7, and are grateful to the editor of *Oncology Letters* for allowing them the opportunity to publish a Corrigendum. Furthermore, they apologize to the readership for any inconvenience caused.

## Figures and Tables

**Figure 7.**
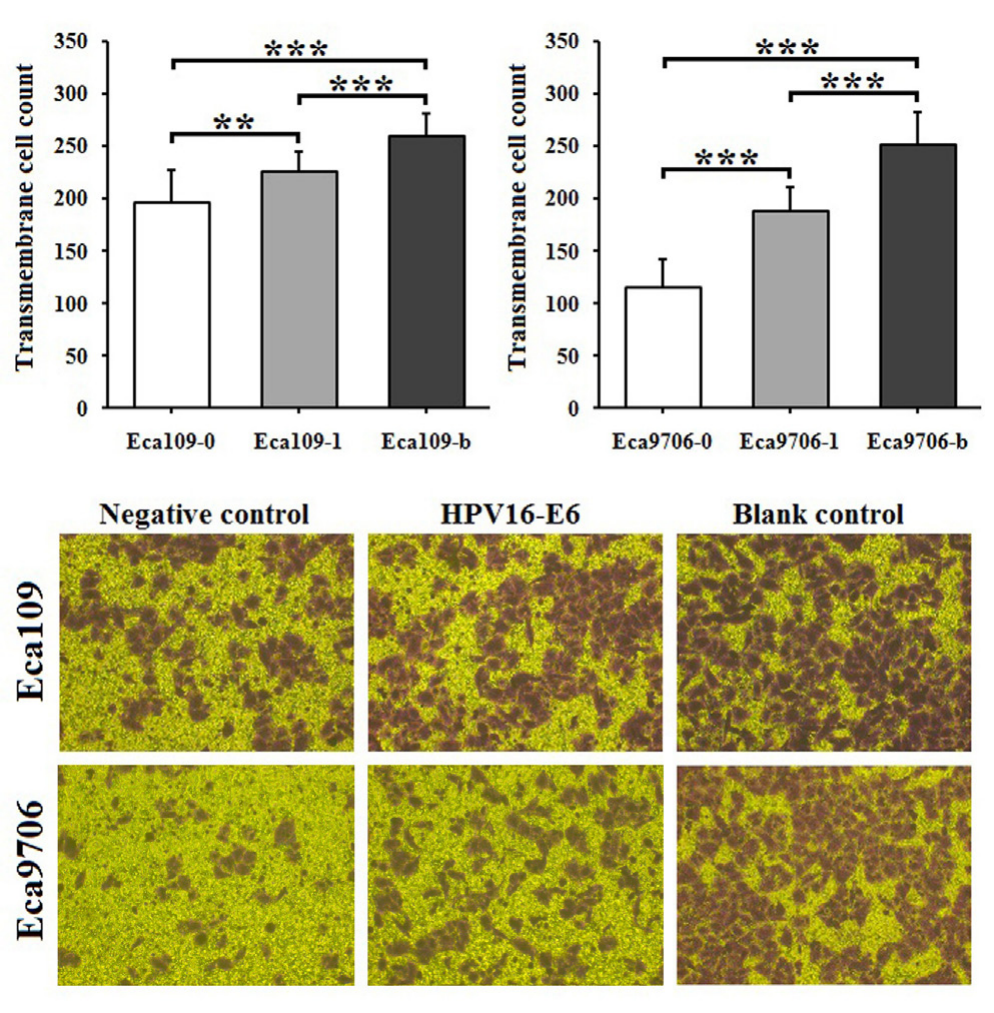
Invasion assay in Eca109 and Eca9706 cells. Magnification, ×100. **P<0.01 and ***P<0.001. −0, transfected with negative control pcDNA3.1; −1, transfected with HPV16E6-pcDNA3.1; −b, blank control; HPV16E6, human papillomavirus type 16 E6 protein.

